# Experimental Study on Grinding of Inner Raceway of Tapered Roller Bearing Outer Ring

**DOI:** 10.3390/mi17020175

**Published:** 2026-01-28

**Authors:** Yingqi Hou, Jiahao Xu, Ziyue Hei, Guangdi Jin, Yufei Gao

**Affiliations:** 1 Key Laboratory of High Efficiency and Clean Mechanical Manufacture of Ministry of Education, School of Mechanical Engineering, Shandong University, Jinan 250061, China; 2 State Key Laboratory of Advanced Equipment and Technology for Metal Forming, Shandong University, Jinan 250061, China; 3 Shandong Key Laboratory of High Performance Tools and System, Shandong University, Jinan 250061, China

**Keywords:** tapered roller bearing, bearing raceway, grinding, roundness error, surface roughness, orthogonal experiment

## Abstract

Tapered roller bearings are widely employed in mechanical structures such as automotive wheel hub units, transmissions, and machine tool spindles, and they have a direct impact on the performance and stability of the equipment. The shape error and surface quality of the bearing raceway, as its working interface, directly affect its service performance. Grinding is an important process in a machining bearing raceway, and the formed roundness error and surface roughness of a raceway affect the workload of subsequent precision polishing processes. In order to reveal the effect of workpiece rotational speed, grinding wheel linear velocity, and grinding depth on the machining quality of the bearing outer ring inner raceway, single-factor experiments and surface roughness orthogonal experiments were conducted. The results were analyzed for range and variance using surface roughness *R*a as the evaluation index, and we developed a mathematical model using a regression method for *R*a. It has been found that the roundness error and surface roughness of the bearing raceway are improved with the increase in the grinding wheel linear velocity and the decrease in the grinding depth and workpiece rotational speed. The grinding depth has the greatest impact on surface roughness and the most significant effect. Next are the grinding wheel linear velocity and the workpiece rotational speed, while the effect of changes in workpiece rotational speed on roughness is relatively insignificant. The lowest surface roughness obtained under the optimized grinding parameter combination is 0.205 μm.

## 1. Introduction

Tapered roller bearings are widely employed in mechanical structures such as automotive wheel hub units, transmission systems, and machine tool spindles. They are suitable for complex working conditions that simultaneously bear radial and axial loads, and they have a direct impact on the property and reliability of equipment [1,2,3]. As the core working interface of bearings, the raceway directly affects key performance indicators such as the bearing capacity, friction characteristics, and fatigue life [4,5,6]. The machining process of a bearing raceway generally goes through stages such as forging, turning, heat treatment, grinding, and precision polishing. Among them, it is a key process to ensure the dimensional accuracy of the raceway [6,7]. Ultra precision machining is the final process, which further improves the surface quality by polishing the raceway with fine-grained oilstone [8,9,10]. The ground surface quality of the bearing ring raceway, such as surface roughness, significantly affects the workload of subsequent precision polishing processes and directly affects processing costs [11,12].

The ground quality of the bearing raceway is a widely concerning issue. Researchers have conducted extensive research on grinding processes, surface roughness, and dimensional accuracy. For a grinding bearing raceway, the surface roughness is the most widely concerning issue, and the roundness error of the bearing ring raceway has also received attention from researchers. The roundness error of bearing rings is the deviation between the contour of the ring cross-section and the ideal circle, usually expressed as the maximum radius difference. It directly affects the rotational accuracy, sealing performance, lubrication effect, and equipment stability of the bearing, and it may lead to premature failure or system failure of the bearing. In terms of process parameters that affect the quality of grinding processing, they mainly involve the grinding wheel linear velocity, grinding depth, and bearing ring rotational speed. Li et al. [13] used a CNC grinder and diamond grinding wheels to conduct grinding experiments to analyze the influence of grinding parameters on the ground quality of ZrO_2_ and Si_3_N_4_ bearing outer rings. And the experimental results indicated that proper grinding parameters could improve roughness (Ra) and the roundness tolerance of the outer ring raceway. Li et al. [14] found in further experimental studies that the *R*a value of the ceramic ring increases with the increase in grinding wheel feed rate and the decrease in the grinding wheel speed. Jin et al. [15] established a prediction model for the surface roughness of the inner raceway grinding process of tapered roller bearings, and conducted a single-factor analysis on the influence of grinding parameters on the working surface roughness of the raceway. The study found that all factors have an impact on the surface roughness. Chi et al. [16] established a new material removal rate model in grinding based on the bearing ring surface shape analysis, which provides a reference for the processing of bearing rings. Chivu et al. considered the inner and outer diameters, width and weight, machined surface roughness, grinding wheel speed, cutting speed, feed rate, and cutting depth, and they used the Holistic Optimization Method (HOM) to predict the grinding time [17]. Li et al. aimed to improve the dressing accuracy of the grinding wheel from the perspective of improving the dressing process, thereby ensuring the quality of grinding [18]. In addition, scholars have conducted a series of studies on the state monitoring [19,20], modeling and simulation [21], grinding efficiency optimization [22], and grinding process design [23,24] in a grinding bearing ring raceway.

Scholars also focused on developing new grinding processes or hybrid grinding processes to improve the grinding quality. Raine adopted a compensative grinding to manufacture a bearing mounting shaft to minimize the installed bearing inner ring roundness error [25]. Regarding the grinding of a bearing ring, Liu et al. [26] proposed an ultra-high-speed centrifugal grinding process, and the bearing ring was prestressed by using a prestress clamping device to reduce the grinding temperature. The corrosion resistance of the bearing ring surface processed by strengthened grinding technology has been improved, but this technology requires higher grinding requirements [27]. Hybrid grinding technology has been a research hotspot in recent years. Ultrasonic vibration-assisted grinding can improve the grinding quality and accuracy of a bearing ring raceway [28,29,30], but this process increases the complexity for application in industry. Both electrolytic grinding [30] and electrochemical grinding [31] can improve the grinding effect, but they increase the complexity of the grinding process and are not widely used in the actual engineering production of a grinding bearing ring. Existing research results have shown that the grinding process parameters of bearing ring raceways can affect their surface quality and shape accuracy. Selecting appropriate grinding parameters is crucial for achieving a high-precision and high-quality bearing ring raceway in the commonly used grinding process in engineering production, which is of great significance for improving the grinding process of bearing ring raceways and reducing the cost of subsequent precision finishing processes. Chang et al. [32] designed the orthogonal experiment to study the discrete degree of grinding surface integrity of a bearing raceway and select the optimal grinding parameters. The Design of Experiments (DoE) method is widely adopted in various machining processes to analyze the influence and optimize process parameters, such as diamond wire sawing [33], grinding [34], micro milling [35], and laser machining [36].

The inner raceway of a TR0708 tapered roller bearing outer ring was taken as the research object of grinding processing. When grinding the raceway of such bearings, the rotational axis of the grinding wheel is not parallel to the rotational axis of the bearing ring, which is different from the common inner circle grinding process. Therefore, the dimensional accuracy and surface quality of its grinding are more worthy of attention. Although theoretical modeling and simulation analysis have been conducted on material removal, grinding force, and grinding heat in the grinding process of the bearing raceway in our previous research work [11,15], there is still a lack of systematic experimental research on grinding parameters. Therefore, in this paper, firstly, the single-factor analysis method was used to conduct grinding experiments on the bearing raceway, focusing on the roundness error and surface roughness. The influence of three main grinding parameters, namely the grinding wheel linear velocity, workpiece rotational speed, and grinding depth, on the roundness error and surface roughness of the inner raceway of the outer ring was studied. Secondly, an orthogonal experiment was designed for further analyzing the surface roughness of the bearing raceway. The experimental results were subjected to range and variance analysis, and a mathematical regression model of surface roughness was established. At last, the optimal grinding parameter combination was determined. The research results can provide an experimental basis and reference for optimizing the grinding process parameters and improving the machining quality of the inner raceway of the outer ring of tapered roller bearings.

## 2. Materials and Methods

### 2.1. Experimental Materials and Equipment

**Figure 1:**
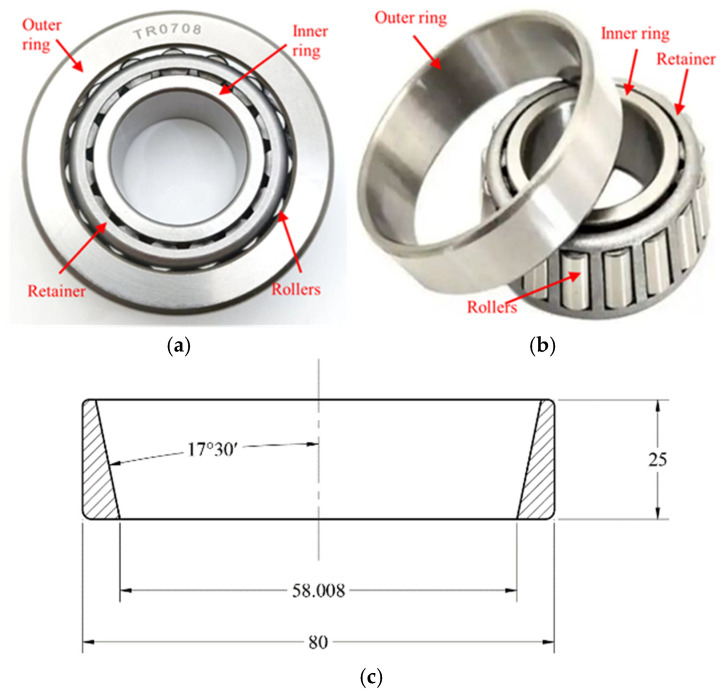
Appearance and outer ring size parameters of TR0708 tapered roller bearing: (**a**) assembled, (**b**) outer ring not assembled, and (**c**) main dimensions of the outer ring.

TR0708 tapered roller bearing (Shandong Chaoyang Bearing Co., Ltd., Dezhou, China) was adopted for grinding, which can withstand both radial and axial loads simultaneously. It is widely used in mechanical structures such as automotive wheel hub units, transmissions, and machine tool spindles. The grinding surface studied in this experiment is the inner raceway of the outer ring, with an inner diameter, outer diameter, width, and tilt angle of Ø 58.008 mm, Ø 80 mm, 25 mm, and 17°30′, respectively. The material used is GCr15 bearing steel. Figure 1 shows the bearing appearance and outer ring dimensions. The bearing inner raceway of the outer ring serves as its working interface, and its shape tolerance and surface roughness directly affect the working performance and rotational accuracy of the bearing.

**Figure 2:**
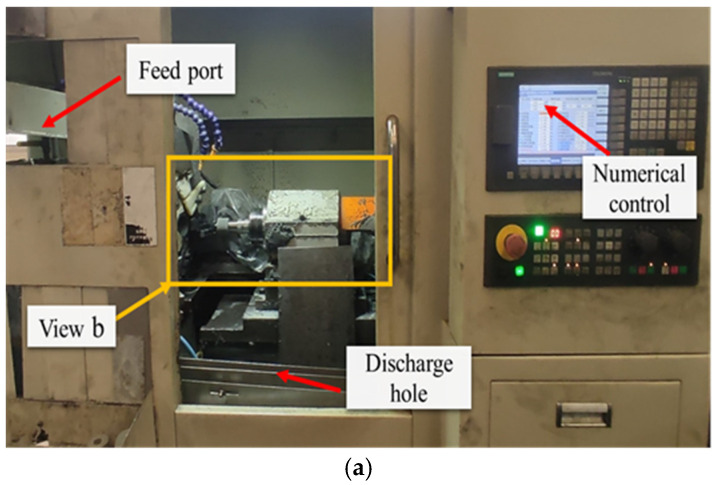

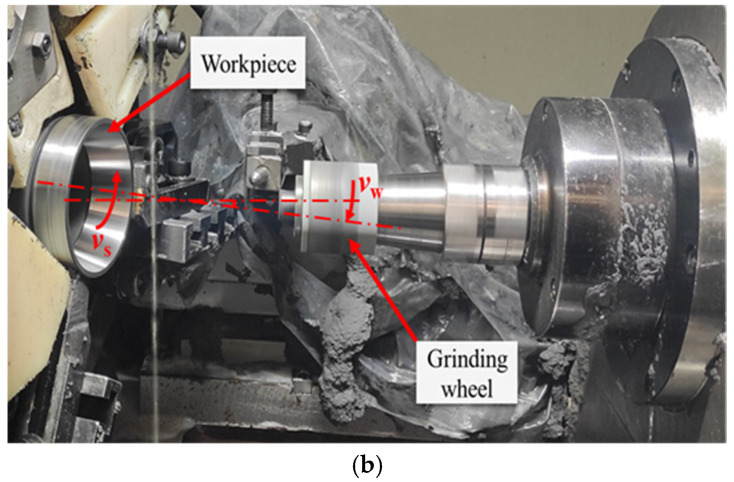
(**a**) Overall appearance of CNC grinder; (**b**) detailed presentation of machining area.

**Figure 3:**
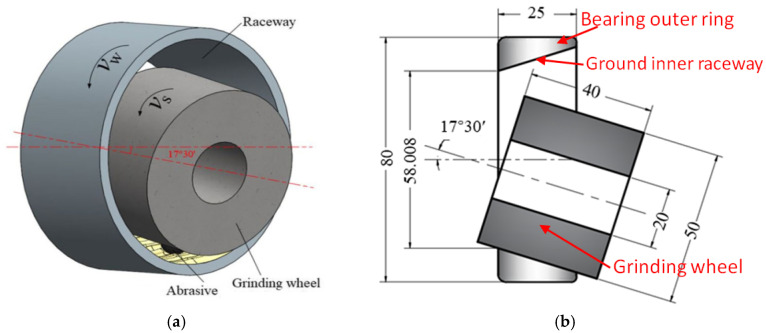
(**a**) Three-dimensional schematic of grinding processing; (**b**) schematic of grinding section of the inner raceway of the outer ring.

The grinding experiment used a CNC grinder made by Pusen Precision Machine Tool Manufacturing Co., Ltd., Wuxi, China. Figure 2a shows the appearance, and Figure 2b presents the details of its machining area. During grinding, a magnetic suction device is used to clamp the bearing outer ring, and the inner circular down grinding method is adopted. Figure 3a,b shows the schematic of the grinding direction, where the bearing outer ring linear velocity is *v*_w_ and the grinding wheel linear velocity is *v*_s_. A ceramic bonded white corundum grinding wheel is selected, with an outer diameter, aperture, and thickness of 50 mm, 20 mm, and 40 mm, respectively. The microstructure number is 7, and the abrasive size is 100 #. The grinding process uses a bearing-specific grinding water-soluble emulsion.

### 2.2. Experimental Design

For grinding of the bearing inner raceway of the outer ring, the grinding wheel linear velocity (*v*_s_), workpiece rotational speed (*n*_w_), and grinding depth (*a*_e_) are the main parameters that affect the roundness error and surface roughness. So, the experimental research is conducted on these three process parameters to study the effect of these main parameters on roundness error and surface roughness of the bearing raceway.

Excessive roundness error can lead to unstable operation and a shortened lifespan of bearings, and it is difficult to correct in the subsequent precision polishing stage. Therefore, single-factor grinding experiments were first designed to study the range of roundness error and the effect of process parameters. A single-factor experimental design is a commonly used statistical experimental design method, which controls the change in one variable while keeping other factors constant to clearly understand the impact of a certain factor on the results. For example, in the experiment, we keep the workpiece rotational speed and grinding depth constant while changing the grinding wheel linear velocity to analyze the impact of this factor on the roughness error of the bearing raceway. The experimental parameters were selected within the range of the CNC grinder, as shown in Table 1.

The bearing raceway surface roughness is also mainly affected by the above three grinding parameters. So, three sets of single-factor grinding experiments are also conducted, which are the same as the experimental parameter design for analyzing the roundness error of bearing raceway grinding, referring to Table 1.

The bearing raceway surface roughness significantly affects the workload of subsequent precision polishing processes. Therefore, taking the minimum surface roughness as the optimization objective in the grinding process and obtaining the optimal grinding parameter combination within the experimental parameter range is of reference value for engineering applications. Therefore, a grinding orthogonal experiment on surface roughness was designed here, using a three-factor, three-level design, and each factor was set at three levels. When selecting the level of orthogonal experimental factors, it is necessary to ensure that the roundness error of the inner raceway of the outer ring meets production requirements. Therefore, it is necessary to select process parameter levels for orthogonal experimental research on surface roughness after analyzing the roundness error of the raceway.

### 2.3. Evaluation of Roundness Error and Ra of Ground Bearing Raceway

**Figure 4:**
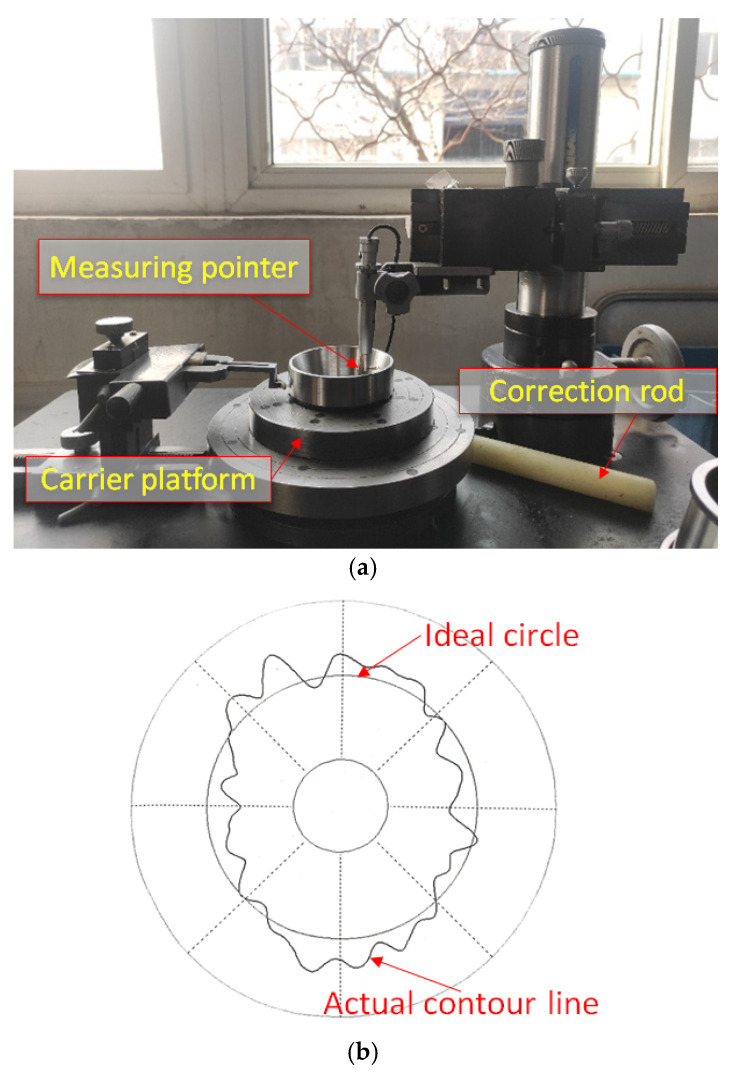
(**a**) Roundness measurement of the bearing inner raceway of the outer ring, and (**b**) schematic diagram of roundness error.

The deviation between the contour of the bearing outer ring raceway cross-section and the ideal circle is the roundness error, which is represented by the maximum radius difference. The roundness error of the ground raceway is measured using a Y90 roundness meter produced by Luoyang Huizhi Control Technology Co., Ltd., Luoyang, China, as shown in Figure 4a, and Figure 4b is a schematic diagram of the occurrence of roundness errors in the raceway. During measurement, the bearing outer ring is placed on the stage of the roundness tester. Turn on the switch to rotate the worktable, and use a calibration rod to accurately align the bearing outer ring with the center of the stage for measurement. After measurement, the roundness meter outputs the roundness error value, and the same raceway is measured 5 times.

**Figure 5:**
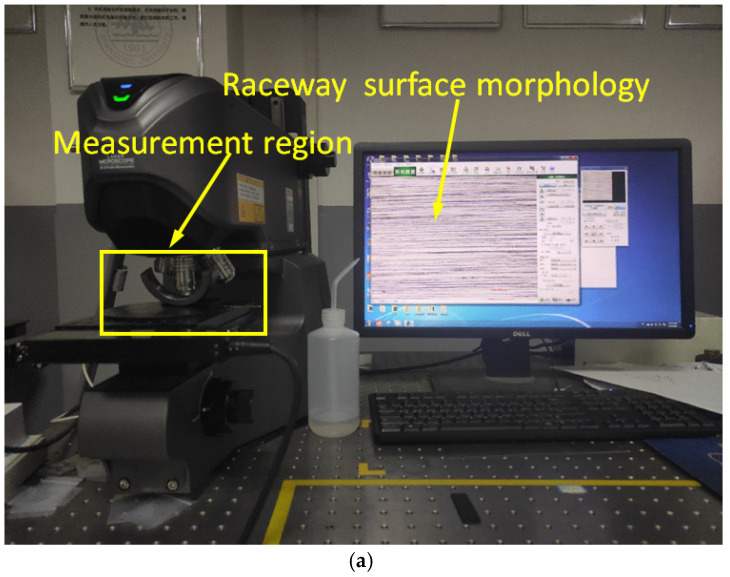

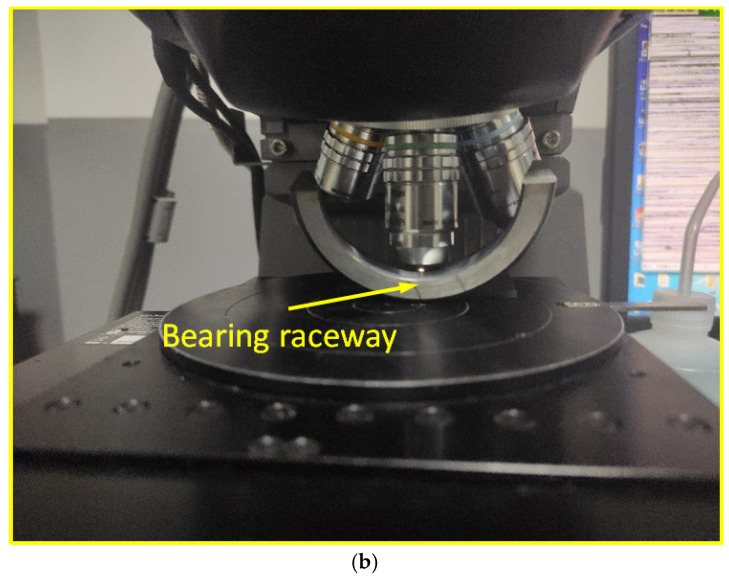
(**a**) Surface roughness measurement of bearing outer race raceway, and (**b**) magnification of measurement area.

The raceway surface roughness is measured using a laser scanning microscope VK-X200K (Keyence Corporation, Osaka, Japan), and the ground surface morphology can also be measured, as shown in Figure 5. After measuring the roundness error of the bearing raceway, an electric discharge wire cutting machine was used to cut the bearing outer ring in the radial direction, three points in the middle of the bearing raceway were selected in the radial direction for measurement, and the average of the measurement results was taken.

## 3. Results and Discussion

### 3.1. Effect of Grinding Parameters on Raceway Roundness Error

**Figure 6:**
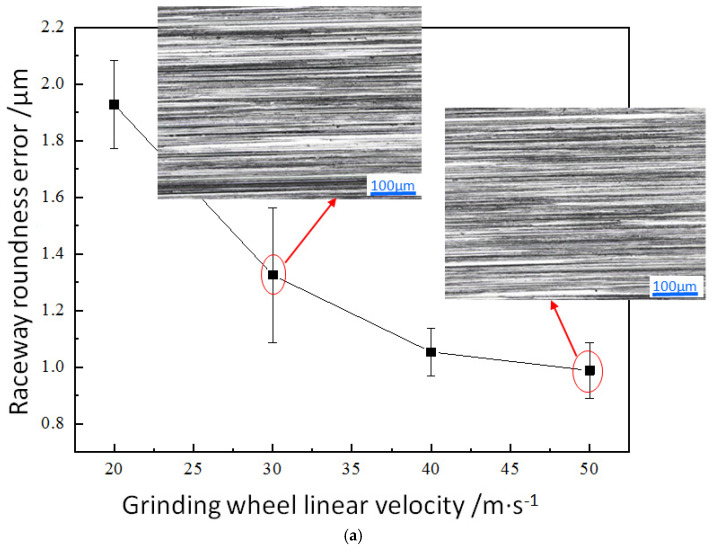

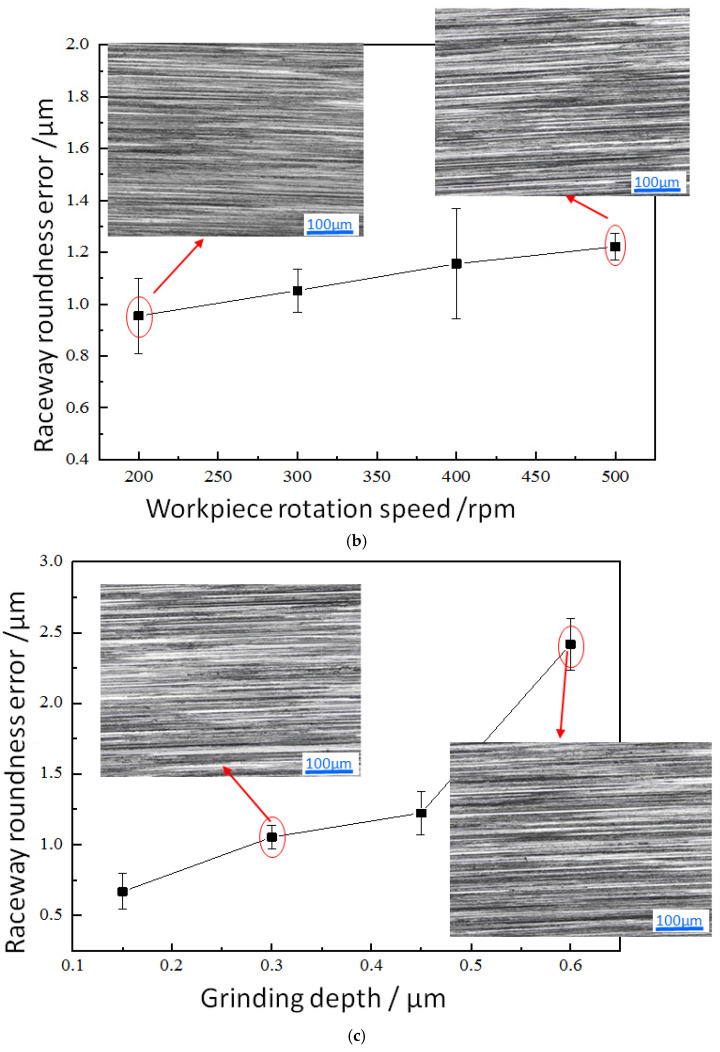
Effect of grinding parameters on raceway roundness error: (**a**) grinding wheel linear velocity (*n*_w_ = 300 rpm, *a*_e_ = 0.3 μm), (**b**) workpiece rotational speed (*v*_s_ = 40 m·s^−1^, *a*_e_ = 0.3 μm), and (**c**) grinding depth (*v*_s_ = 40 m·s^−1^, *n*_w_ = 300 rpm).

The roundness error of the raceway is related to the improvement of cutting characteristics, thermal effects, and dynamic stability during the grinding process. The roundness error of the bearing raceway under different processing parameters is shown in Figure 6. Figure 6a shows the effect of grinding wheel linear velocity on the roundness error of the raceway. When the grinding wheel linear velocity increases from 20 m·s^−1^ to 50 m·s^−1^, the average roundness error decreases from 1.928 μm to 0.988 μm. Increasing the grinding wheel speed can enhance the dynamic stability of the grinding process, reduce the vibration disturbance on the workpiece surface, enhance the dynamic stiffness of the system, and reduce the elastic deformation caused by periodic cutting forces, and thus improve the roundness accuracy of the bearing raceway. A high-speed rotating grinding wheel can remove materials more evenly and reduce the risk of deformation caused by local stress concentration. A higher grinding wheel linear velocity can shorten the heat accumulation time in the contact area between the grinding wheel and the workpiece, reducing the impact of thermal deformation on roundness [37,38]. At the same time, as the grinding wheel linear velocity increases, more and more abrasives participate in grinding per unit time in the grinding arc zone, and the volume of material removed by a single abrasive decreases. When comparing the ground surface morphology when the grinding wheel linear velocity increased from 30 m·s^−1^ to 50 m·s^−1^ (shown in Figure 6a), it can be found that the distribution uniformity of surface grinding marks obtained by grinding at a low grinding wheel linear velocity is poor. With the increase in grinding wheel linear velocity, the number of abrasives participating in grinding per unit time increases, and more abrasive motion trajectories overlap during grinding. The distribution uniformity of surface grinding marks is improved, and the surface quality is improved. Correspondingly, the roundness error is also reduced.

Figure 6b shows the effect of workpiece rotational speed on the roundness error of the raceway. When the workpiece rotational speed increases from 200 rpm to 500 rpm, the average roundness error increases from 0.955 μm to 1.222 μm. Within the range of experimental parameter values, the variation in roundness error values is relatively small, and the difference in surface morphology is not significant. The increase in workpiece rotational speed leads to a decrease in the number of abrasives involved in grinding per unit time, resulting in a slight reduction in the distribution uniformity of surface grinding marks. In addition, when the workpiece rotational speed is 500 rpm, the roundness error bar increases, which also reflects that high-speed rotation of the workpiece may intensify vibration, leading to an increase in roundness error [25]. Therefore, the workpiece rotational speed should not be too high. In addition, from the perspective of grinding temperature, an increase in the ring rotational speed leads to a local temperature rise in the grinding area, and the ring will undergo non-uniform deformation due to thermal expansion, resulting in an increase in the roundness error of the raceway.

Figure 6c shows the effect of grinding depth on the roundness error of the raceway surface. When the grinding depth increases from 0.15 μm to 0.6 μm, the average roundness error increases from 0.668 μm to 2.417 μm. When the grinding depth is small, the number of effective abrasives involved in grinding is relatively small, the maximum undeformed chip thickness is small, the material removal rate is small, there is less material accumulation and more evenly distributed grinding marks on the ground surface, the raceway surface is relatively flat, and the roundness error is small. Existing research results indicate that as the grinding depth increases, the grinding force and temperature also increase. Under these working conditions, a dark layer may be generated on the ground surface, which may manifest as microcracks, residual stresses, structural changes, or work hardening. The dark layer can affect the dimensional stability of the workpiece, causing deformation due to residual stress release or subsequent processing, and affecting the grinding dimensional accuracy [37,38].

From the above experimental results, it can be seen that when the grinding wheel linear velocity is small and the grinding depth is large, the roundness error of the raceway is relatively large. In the grinding experiment conducted with the adopted process parameter combination, the range of roundness error of the raceway was between 0.668 and 2.417 μm. For this type of bearing, the production requirement is that the roundness error of the raceway should generally be less than 1.5 μm. Therefore, when determining the factor level of a surface roughness orthogonal experiment, the grinding wheel linear velocity of 20 m·s^−1^ and the grinding depth of 0.6 μm are not used, and the stability of the workpiece operation during the grinding process is considered. When determining the three levels of workpiece speed in the orthogonal experiment, 500 rpm is also not used.

### 3.2. Effect of Grinding Parameters on Surface Roughness

**Figure 7:**
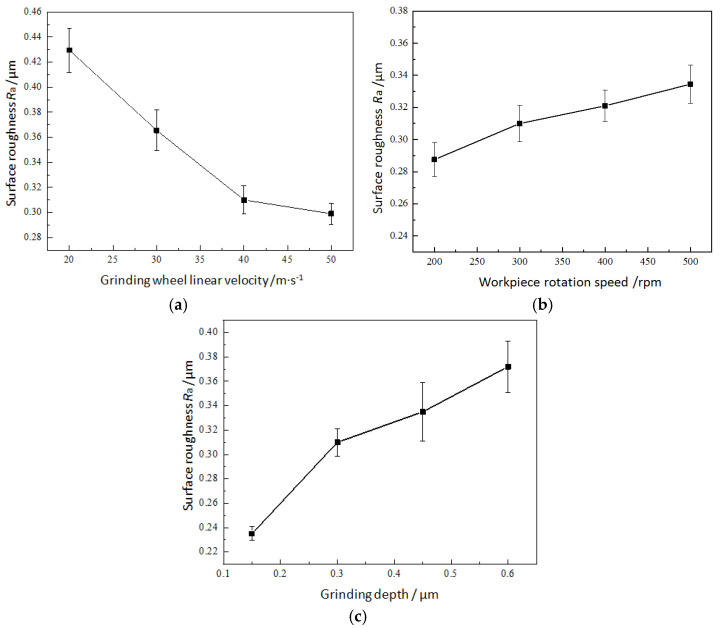
Effect of grinding parameters on surface roughness: (**a**) grinding wheel linear velocity (*n*_w_ = 300 rpm, *a*_e_ = 0.3 μm), (**b**) workpiece rotational speed (*v*_s_ = 40 m·s^−1^, *a*_e_ = 0.3 μm), and (**c**) grinding depth (*v*_s_ = 40 m·s^−1^, *n*_w_ = 300 rpm).

The influence of grinding parameters on the raceway surface roughness was analyzed using the experimental parameter combination design shown in Table 1, as shown in Figure 7. Figure 7a shows the influence of grinding wheel linear velocity on the raceway surface roughness. When the grinding wheel linear velocity decreases from 50 m·s^−1^ to 20 m·s^−1^, the average surface roughness increases from 0.299 μm to 0.430 μm. An increase in the grinding wheel linear velocity can remove materials more evenly to improve the surface morphology and make the surface ground texture more regular and evenly distributed (as shown in Figure 6a), resulting in a surface roughness decrease.

Figure 7b shows the variation in the raceway surface roughness at different workpiece rotational speeds. When the workpiece rotational speed increases from 200 rpm to 500 rpm, the average surface roughness increases from 0.288 μm to 0.335 μm. The increase in workpiece rotational speed reduces the degree of coincidence of abrasive trajectories. In addition, with the same grinding depth, increasing the workpiece rotational speed also leads to an increase in the maximum undeformed chip thickness during grinding, resulting in a surface roughness increase.

Figure 7c shows the influence of grinding depth on the raceway surface roughness. The average surface roughness value decreases from 0.372 μm to 0.235 μm, while the grinding depth decreases from 0.6 μm to 0.15 μm. That is, when the grinding depth decreases, the raceway surface roughness decreases. Based on the surface morphology obtained at grinding depths of 0.3 μm and 0.6 μm, as shown in Figure 6c, it can be observed that a smaller grinding depth can result in a relatively smooth grinding surface. As the grinding depth increases, the contact area between the abrasives in the grinding arc zone increases. Due to the normal distribution characteristics of abrasive protrusions’ height on the grinding wheel, the height differences in grinding marks formed by the adjacent abrasives removing the material become relatively larger, which also increases the surface roughness value.

### 3.3. Orthogonal Experimental Design and Result Analysis

The bearing raceway surface roughness significantly affects the workload of subsequent precision polishing processes. The grinding process aims to achieve low surface roughness, to reduce the difficulty of subsequent precision polishing processes. Therefore, an orthogonal experiment on surface roughness is designed. Orthogonal experimental design is a method of scientifically arranging and analyzing multi-factor experiments using orthogonal tables. Its core idea is to screen representative horizontal combinations through orthogonality, so that experimental points have the characteristics of “uniform dispersion, neatness and comparability”, while ensuring efficiency and reducing the resource consumption of comprehensive experiments. The research objective is to determine the primary and secondary effects of various grinding process parameters on the surface roughness of a bearing raceway, and to obtain the optimal parameter combination within the experimental parameter range. Based on the analysis results of the roundness error of the raceway in Section 3.1, the three levels of each factor selected are shown in Table 2, and the corresponding combination design of factors and levels is shown in Table 3. Compared to traditional factorial design, orthogonal experiments significantly reduce the number of experiments, and the three-factor, three-level experiment can be reduced from 27 comprehensive experiments to 9 experiments. However, this experimental design lacks sufficient handling of the interaction between multiple grinding process parameters.

Table 4 shows the surface roughness values obtained by grinding with various parameter combinations in the orthogonal experiment. The range analysis results based on the surface roughness results in Table 4 are shown in Table 5. Range refers to the difference between the minimum and maximum average values of the indicators corresponding to each level in each column. If the range of a column is larger, it indicates that the change in the value of the experimental indicator within the experimental range is greater, that is, the greater the impact on the experimental indicator. According to the analysis results in Table 5, the range values of the influence of grinding wheel linear velocity, workpiece rotational speed, and grinding depth on surface roughness are 0.074, 0.037, and 0.099, respectively. So, the order of the influence of various grinding parameters on the raceway surface roughness from high to low is grinding depth, grinding wheel linear velocity, and workpiece rotational speed. Previous studies on the temperature field and grinding dark layer of GCr15 quenched bearing steel have found that the influence of grinding depth is the most significant compared to grinding wheel linear velocity and workpiece rotational speed [11]. The dark layer structure is mainly composed of tempered martensite, tempered martensite, and tempered martensite, and the material properties are uneven [37,38]. When grinding the dark layer, the surface plastic deformation and abrasive scratches deepen, deteriorating the surface roughness.

The significant effects of grinding wheel linear velocity, workpiece rotational speed, and grinding depth on surface roughness can be obtained through variance analysis, as shown in Table 6. The analysis results show that the *F* value of the grinding depth is 19.082, indicating that the change in grinding depth has a significant impact on surface roughness. The *F* value of the grinding wheel linear velocity is 10.755, which has a quite significant impact on surface roughness, while the *F* value of the workpiece rotation speed is 2.665, indicating that changes in workpiece rotation speed have no significant effect on roughness.

By conducting regression analysis on the grinding parameters and surface roughness, a regression calculation equation of surface roughness can be obtained as follows:

*R*a = 0.868 × *v*_s_^−0.429^ × *n*_w_^0.157^ × *a*_e_^0.289^ (1)

From the above equation, it can be seen that the grinding wheel linear velocity *v_s_* has a negative effect on the surface roughness *R*a, while the workpiece rotational speed *n*_w_ and grinding depth *a*_e_ have a positive effect on the surface roughness *R*a, which is consistent with the analysis results of single-factor experiments. After calculation, the R^2^ of this regression model is 0.938. R^2^ (R-squared) is an indicator that measures the fit degree of a regression model, with a range of values from 0 to 1. The larger the R^2^ value, the better the regression model. The regression equation can be employed to calculate the ground surface roughness within the experimental parameter range. It is worth noting that to further improve the calculation accuracy of the regression model, it is necessary to increase experimental data, optimize regression models, or adopt advanced machine learning methods.

Based on the analysis results in Table 5, the combination *A*_3_*B*_1_*C*_1_ is the optimal parameter combination for the surface roughness experiment of bearing raceway grinding; that is, the grinding wheel linear velocity is 50 m·s^−1^, the workpiece rotational speed is 200 rpm, and the grinding depth is 0.15 μm. Since the obtained optimal combination of grinding parameters did not appear in the orthogonal experiment, the bearing raceway was machined using these grinding parameters to verify the correctness of the analysis. The surface roughness average value measured by ground with the optimized processing parameter combination is 0.205 μm. The surface roughness value obtained by calculating the combination of grinding parameters using equation 1 is 0.215 μm, which is very close to the experimental value, with a relative error of 4.878%, indicating that the regression model has a reasonable calculation accuracy. The obtained value is smaller than the surface roughness values under other processing parameters in single-factor experiments and orthogonal experiments, which is consistent with the analysis results of orthogonal experiments.

## 4. Conclusions

This paper used a single-factor experimental method to analyze the influence of the main processing parameters such as the grinding wheel linear velocity, workpiece rotational speed, and grinding depth on the roundness error and surface roughness of the inner raceway of the outer ring of tapered roller bearings. Orthogonal experiments were designed to obtain the primary and secondary relationships of the influence of various process parameters on surface roughness through range analysis and variance analysis. The optimal combination of process parameters for grinding bearing raceway surface roughness was obtained, and the following conclusions were drawn:

1. When using a low grinding wheel linear velocity and large grinding depth, the surface morphology obtained by grinding shows a poor distribution uniformity of grinding marks. Within the range of experimental parameter values, the effect of changes in workpiece rotational speed on surface morphology is not significant. However, a high workpiece rotational speed reduces the uniformity of surface grinding marks’ distribution.
2. The roundness error and surface roughness of the raceway are improved with the increase in grinding wheel linear velocity and the decrease in workpiece rotational speed and grinding depth. A lower grinding wheel linear speed (20 m·s^−1^) and larger grinding depth (0.6 μm) can lead to roundness errors in the raceway exceeding the machining requirements.
3. The grinding depth has the greatest impact on surface roughness and the most significant effect, followed by the grinding wheel linear velocity and workpiece rotational speed, while the effect of workpiece rotational speed changes on surface roughness is relatively insignificant. Based on the grinder used in this experiment, within the range of process parameters studied, the surface roughness of the inner raceway of the outer ring of the tapered roller bearing obtained is the lowest at 0.205 μm when the grinding wheel linear velocity is 50 m·s^−1^, the workpiece rotational speed is 200 rpm, and the grinding depth is 0.15 μm.

The research results provide a basis for understanding the influence of grinding process parameters on the grinding quality of tapered roller bearings and optimizing process parameters, which can serve as a reference for engineers in this field. However, other experimental design methods such as the response surface methodology are needed to further explore the interactions between these process parameters and their effects on the surface properties of the ground bearing raceway.

## Figures and Tables

**Table 1:** Single-factor experimental design for analysis of grinding roundness error of the inner raceway the outer ring.

Parameters	Group 1	Group 2	Group 3
Grinding wheel linear velocity/m·s^−1^	20, 30, 40, 50	40	40
Workpiece rotational speed/rpm	300	200, 300, 400, 500	300
Grinding depth/μm	0.3	0.3	0.15, 0.3, 0.45, 0.6

**Table 2:** Levels of various factors in orthogonal experiment of surface roughness.

Level	Factors		
	(*A*) Grinding Wheel Linear Velocity *v*_s_/m·s^−1^	(*B*) Workpiece Rotational Speed *n*_w_/rpm	(*C*) Grinding Depth *a*_e_/μm
1	30 (*A*_1_)	200 (*B*_1_)	0.15 (*C*_1_)
2	40 (*A*_2_)	300 (*B*_2_)	0.3 (*C*_2_)
3	50 (*A*_3_)	400 (*B*_3_)	0.45 (*C*_3_)

**Table 3:** Orthogonal experimental factors and levels’ combination design for surface roughness.

Number	Grinding Parameter Combination		
1	*A*_1_	*B*_1_	*C*_1_
2	*A*_2_	*B*_2_	*C*_2_
3	*A*_3_	*B*_3_	*C*_3_
4	*A*_1_	*B*_2_	*C*_3_
5	*A*_2_	*B*_3_	*C*_1_
6	*A*_3_	*B*_1_	*C*_2_
7	*A*_1_	*B*_3_	*C*_2_
8	*A*_2_	*B*_1_	*C*_3_
9	*A*_3_	*B*_2_	*C*_1_

**Table 4:** Surface roughness measurement results of orthogonal experiment.

Number	*v*_s_/m·s^−1^	*n*_w_/rpm	*a*_e_/μm	*R*a/μm
1	30	200	0.15	0.290
2	40	300	0.3	0.310
3	50	400	0.45	0.351
4	30	300	0.45	0.380
5	40	400	0.15	0.268
6	50	200	0.3	0.235
7	30	400	0.3	0.357
8	40	200	0.45	0.341
9	50	300	0.15	0.218

**Table 5:** Range analysis results of surface roughness.

Direct Analysis	Factors		
	*v*_s_/m·s^−1^	*n*_w_/rpm	*a*_e_/μm
*K*_1_	1.027	0.866	0.776
*K*_2_	0.919	0.908	0.902
*K*_3_	0.804	0.976	1.072
*k*_1_	0.342	0.289	0.259
*k*_2_	0.306	0.303	0.301
*k*_3_	0.268	0.325	0.357
*R*	0.074	0.037	0.099

**Table 6:** Significance analysis of processing parameters on surface roughness.

Factor	Deviation	Degrees of Freedom	Mean Square Deviation	*F*	Significance
*A*	0.008	2	0.004	10.755	Quite significant impact
*B*	0.002	2	0.001	2.665	No significant impact
*C*	0.015	2	0.007	19.082	Significant impact
Error	0.001	2	0.001		
Sum	0.026	8			

## Data Availability

The original contributions presented in this study are included in the article. Further inquiries can be directed to the corresponding author.
